# INSPIRA: study protocol for a randomized-controlled trial about the effect of spirometry-assisted preoperative inspiratory muscle training on postoperative complications in abdominal surgery

**DOI:** 10.1186/s13063-022-06254-4

**Published:** 2022-06-07

**Authors:** D. L. Birrer, C. Kuemmerli, A. Obwegeser, M. Liebi, S. von Felten, K. Pettersson, K. Horisberger

**Affiliations:** 1 Department of Transplantation and Surgery, University Hospital of Zurich, Zurich, Switzerland; 2 Department of Surgery, Clarunis-University Centre for Gastrointestinal and Liver Diseases Basel, Basel, Switzerland; 3 Department of Physiotherapy and Occupational Therapy, University Hospital of Zurich, Zurich, Switzerland; 4 Department of Biostatistics, Epidemiology and Prevention Institute, University of Zurich, Zurich, Switzerland; 5 Department of Surgery and Transplantation, University Hospital Mainz, Mainz, Germany

**Keywords:** Respiratory physiotherapy, Respiratory complications, Abdominal surgery, Preoperative, Inspiratory muscle training, Pre-habilitation, Postoperative complications, Overall morbidity

## Abstract

**Background:**

Rehabilitation strategies after abdominal surgery enhance recovery and improve outcome. A cornerstone of rehabilitation is respiratory physiotherapy with inspiratory muscle training to enhance pulmonary function.

Pre-habilitation is the process of enhancing functional capacity before surgery in order to compensate for the stress of surgery and postoperative recovery. There is growing interest in deploying pre-habilitation interventions prior to surgery.

The aim of this study is to assess the impact of preoperative inspiratory muscle training on postoperative overall morbidity. The question is, whether inspiratory muscle training prior to elective abdominal surgery reduces the number of postoperative complications and their severity grade.

**Methods:**

We describe a prospective randomized-controlled single-centre trial in a tertiary referral centre. The primary outcome is the Comprehensive Complication Index (CCI) at 90 days after surgery. The CCI expresses morbidity on a continuous numeric scale from 0 (no complication) to 100 (death) by weighing all postoperative complications according to the Clavien-Dindo classification for their respective severity.

In the intervention group, patients will be instructed by physiotherapists to perform inspiratory muscle training containing of 30 breaths twice a day for at least 2 weeks before surgery using Power®Breathe KHP2. Depending on the surgical schedule, training can be extended up to 6 weeks. In the control group, no preoperative inspiratory muscle training will be performed. After the operation, both groups receive the same physiotherapeutic support.

**Discussion:**

Existing data about preoperative inspiratory muscle training on postoperative complications are ambiguous and study protocols are often lacking a clear design and a clearly defined endpoint. Most studies consist of multi-stage concepts, comprehensively supervised and long-term interventions, whose implementation in clinical practice is hardly possible. There is a clear need for randomized-controlled studies with a simple protocol that can be easily transferred into clinical practice. This study examines the effortless adjustment of the common respiratory physiotherapy from currently postoperative to preoperative. The external measurement by the device eliminates the diary listing of patients’ performances and allows the exercise adherence and thus the effect to be objectively recorded.

**Trial registration:**

ClinicalTrials.govNCT04558151. Registered on September 15, 2020.

## Administrative information

Note: the numbers in curly brackets in this protocol refer to SPIRIT checklist item numbers. The order of the items has been modified to group similar items (see http://www.equator-network.org/reporting-guidelines/spirit-2013-statement-defining-standard-protocol-items-for-clinical-trials/).

**Table:** 

Title {1}	INSPIRA: study protocol for a randomized-controlled trial about the effect of spirometry-assisted preoperative inspiratory muscle training on postoperative complications in abdominal surgery
Trial registration {2a and 2b}.	ClinicalTrials.gov Identifier: NCT04558151
Protocol version {3}	Version 1.0; August 3^rd^ 2020
Funding {4}	Lunge Zurich
Author details {5a}	DLB^1^ is the Chief Investigator/Principal Investigator; she led the proposal, contributed to study design, led protocol development, wrote the study protocol and conducts the trial at the University Hospital Zurich. ^1^Department of Transplantation and Surgery, University Hospital of Zurich, Switzerland. CK^2^ contributed to study design and to development of the proposal. ^2^Department of Surgery, Clarunis-University Centre for Gastrointestinal and Liver Diseases Basel, Basel, Switzerland. OA^3^ and LM^3^ are physiotherapist, contributed to study design. ^3^Department of Physiotherapy and Occupational Therapy, University Hospital Zurich, Zurich, Switzerland. SvF^4^ was trial statistician. ^4^Department of Biostatistics, Epidemiology, Biostatistics, and Prevention Institute, University of Zurich, Zurich, Switzerland. KP^1^ is the study nurse, contributed to the study design.^1^Department of Transplantation and Surgery, University Hospital of Zurich, Switzerland. KH^1,5^ is the Sponsor; she conceived the study, led the proposal and protocol development. ^1^Department of Transplantation and Surgery, University Hospital of Zurich, Switzerland. ^5^Department of Surgery and Transplantation, University Hospital Mainz. All authors read and approved the final manuscript.
Name and contact information for the trial sponsor {5b}	Karoline Horisberger Klinik für Viszeral- und Transplantationschirurgie Universitätsspital Zürich Rämistrasse 100 CH-8091 Zürich Karoline.horisberger@usz.ch
Role of sponsor {5c}	KH has the ultimate authority about: financial responsibility, study design, protocol development; interpretation of data; report writing.

## Introduction

### Background and rationale {6a}

Postoperative pulmonary complications are frequent and have a significant impact on patient morbidity and mortality. These complications are particularly common after major abdominal procedures and can be considerable. The connection is likely that major abdominal surgery temporarily reduces respiratory function due to reduction of diaphragmatic strength caused by pain, anaesthesia and inhibitory reflexes as a consequence of splanchnic organ manipulation [1]. This reduction may be adequate to lead to complications or to not be able to ward off infections sufficiently. Pulmonary complications increase not only the length of hospital stay, medical consumption, thus suffering and costs [2–4], they are also the main reason for readmissions to critical care units after major surgery [5].

Inspiratory training is a cornerstone of postoperative physiotherapy and has been performed routinely for a long time [6]. The purpose of all inspiratory muscle training in any exercise regimen is to strengthen the muscles and increase breathing volume, thus leading to better physiological reserve. By inspiring against individual resistance, the muscles progressively strengthen by regular training, which may result in improved respiratory capacity [7]. In order to improve the reserves even before an operation—in the consideration of being more resistant with better reserves—physiotherapeutic regimes and studies are increasingly shifting from post- to preoperative [8, 9].

Most studies consist of multiple-component structured programs [10] including nutritional adaptations, high-intensity interval training, and supervised training sessions [11, 12]. The training span of different prehabilitation regimens is wide with a range from one to 6 weeks [13, 14]. However, implementation in clinical practice of these complex training schedules is hardly possible.

While systematic reviews have shown an advantage of these multi-step concepts regarding hospital length of stay, studies evaluating exclusively specific respiratory components are scarce [15, 16].

Recently, a systematic review has attempted to generate evidence about preoperative inspiratory muscle training [17] but the validity of the data remains controversial [18]. To date, there is no evidence from randomized-controlled trials to provide sufficient conclusions. There is a clear need for randomized-controlled studies with a simple protocol that can be easily transferred into clinical practice. Moreover, studies including different lengths of prehabilitation interventions are needed to investigate the impact of intervention duration [19].

Our study examines the effortless adjustment of the common respiratory physiotherapy from currently postoperative to preoperative. The external measurement by the device eliminates the diary listing of patients' performances and allows the exercise adherence and thus the effect to be objectively recorded.

### Objectives {7}

The aim of the study is to assess the impact of preoperative inspiratory muscle training on postoperative overall morbidity. The question is whether inspiratory muscle training prior to elective surgery reduces the number of postoperative complications and their severity grade.

The primary objective is to show in patients with elective major abdominal surgery that inspiratory muscle training before surgery compared to no pre-operative training reduces the number and severity of postoperative complications, as assessed by the Comprehensive Complication Index.

The secondary objective is to investigate in patients with elective major abdominal surgery whether inspiratory muscle training before surgery compared to no pre-operative training affects: postoperative outcome and physiotherapeutic changes.

### Trial design {8}

This is a prospective randomized-controlled single-centre open-label trial with a 1:1 allocation. The framework of the study is designed to test the superiority of preoperative inspiratory muscle training on postoperative complications in abdominal surgery.

## Methods: participants, interventions and outcomes

### Study setting {9}

The study takes place in a university hospital in Switzerland (Ref: University Hospital Zurich USZ)

### Eligibility criteria {10}

#### Inclusion criteria

- Informed consent as documented by signature.
- Planned abdominal surgery with planned duration > 2 h.
- Planned surgery at least 2 weeks after inclusion at the outpatient clinic.
- Adult patient over 18 years.

#### Exclusion criteria

- Inability to follow the procedures of the study, e.g. due to language problems, psychological disorders, dementia, of the participant.
- Known or suspected non-compliance to the study, drug or alcohol abuse.
- Previous enrolment into the current study.
- Participation in another study with inspiratory muscle training within 30 days preceding or during this study.
- Participation in another study with investigational drugs within 30 days preceding or during this study.
- Planned two-stage hepatectomies (e.g. ALPPS).

### Who will take informed consent? {26a}

The investigators will explain to each participant the nature of the study, its purpose, the procedures involved, the expected duration, the potential risks and benefits and any discomfort it may entail. Each participant will be informed that the participation in the study is voluntary and that he/she may withdraw from the study at any time and that withdrawal of consent will not affect his/her subsequent medical assistance and treatment. Enough time needs to be given to the participant to decide whether to participate or not.

The formal consent of a participant, using the approved consent form, will be obtained before the participant is submitted to any study procedure by one of the investigators.

### Additional consent provisions for collection and use of participant data and biological specimens {26b}

On the consent form, participants will be asked if they agree to use of their data should they choose to withdraw from the trial. Participants will also be asked for permission for the research team to share relevant data with people from the Universities taking part in the research or from regulatory authorities, where relevant. This trial does not involve collecting biological specimens for storage.

## Interventions

### Explanation for the choice of comparators {6b}

It is current standard not to perform any physiotherapeutic intervention or respiratory training before surgery.

The training of inspiratory muscles is the cornerstone of postoperative physiotherapy. The change of this simple but effective treatment to the preoperative setting was driven by the known effect and the simplicity of the intervention. In the postoperative setting, the use of pressure threshold inspiratory muscle training devices is meanwhile well-established in clinical practice.

The intervention regimen follows physiotherapeutic standards and instructions are given by a specialized physiotherapist. No additional training of physiotherapists is required due to the fact that the preoperative training is identical with the postoperative one. Training of inspiratory muscles can be performed in an outpatient setting. The training session is performed unsupervised, during the period of preoperative training, a reassessment of the physiotherapist will be undertaken to ensure adherence to training. Two weeks of preoperative training is the assumed minimum to see an effect. At the same time, this interval is very usual in a clinical routine; a longer training would maybe show a more pronounced effect but could probably never be implemented. The possibility of its implementation in daily routine is main reason why we decided to examine a simple procedure in a manageable time frame.

To perform 30 breath twice a day is the usual practice of POWER®breathe.

### Intervention description {11a}

Once patients give their informed consent to the study and are assigned to the intervention group, physiotherapists will measure the maximal inspiration pressure (MIP) with the POWER®breathe device of each single patient. Patients will then be instructed to perform inspiratory muscle training at the level of 60% of their MIP. Patients are instructed to perform the training containing 30 breaths twice a day for 14–18 days before surgery. After 7 days of training, a physiotherapeutic control will be performed to check compliance and training performance and to answer open questions. The devices are able to register the performance of each single training session and training results are monitored.

### Criteria for discontinuing or modifying allocated interventions {11b}

Patients are withdrawn from the study when they withdraw their informed consent. There will be no special criteria for discontinuing or modifying allocated interventions.

### Strategies to improve adherence to interventions {11c}

The POWER®breathe KHP2 devices are able to register the performance of each single training session so that all training results are stored. Furthermore, we are calling the patient on a regular basis, to improve adherence or to detect potential problems. Patients will not have to keep up-to-date records of their training.

### Relevant concomitant care permitted or prohibited during the trial {11d}

There are no specific interventions that would be prohibited during the trial except participation in another study that could interfere with the inspiratory muscle training.

### Provisions for post-trial care {30}

Insurance is covered by “Versicherung für klinische Versuche und nichtklinische Versuche” by Zurich Versicherungs-Gesellschaft AG.

Any damage developed in relation to study participation is covered by this insurance. So as not to forfeit their insurance cover, the participants themselves must strictly follow the instructions of the study personnel. Participants must not be involved in any other medical treatment without permission of the principal investigator (emergency excluded). Medical emergency treatment must be reported immediately to the investigator. The investigator must also be informed instantly, in the event of health problems or other damages during or after the course of study treatment.

The investigator will allow delegates of the insurance company to have access to the source data/documents as necessary to clarify a case of damage related to study participation. All involved parties will keep the patient data strictly confidential.

A copy of the insurance certificate will be placed in the Investigator’s Site File.

### Outcomes {12}

The primary outcome is postoperative complications measured by the Comprehensive Complication Index (CCI) at 90 days after surgery [20, 21]. The CCI expresses postoperative morbidity on a continuous numeric scale from 0 (no complication) to 100 (death) by weighing all postoperative complications according to the validated Clavien-Dindo classification [22] for their respective severity [23]. It is measured at the end-of hospitalization and 90 days after surgery to include cases in which readmission was necessary. Postoperative complications are the gold standard in the evaluation of the quality of surgery as they directly reflect the procedures outcome and are most relevant for the patients.

Secondary endpoints are postoperative morbidity along the classification of Clavien-Dindo at the end of hospitalization as well as 90 days +/− 2 weeks after surgery [22, 23]. We will further analyse major complications ≥3b after 90 days. This classification consists of five (1–5) severity grades. Grade 1 reflects minor complications, while grade 5 reflects death. The classification is widely accepted and validated to report postoperative complications. It is also reported at the end of hospitalization as well as 90 days after surgery.

Further secondary endpoints are length of hospital stay (LOS), readmission rate (during 90 days) and mortality (during 90 days) as a reflection of postoperative outcome.

Physiotherapeutic values are the MIP, LOAD, POWER, and Breathing Energy. MIP is the most commonly used measure to evaluate inspiratory muscle strength. Very sensitive in detecting early respiratory muscle dysfunction, it allows for the assessment of ventilatory failure, restrictive lung disease and respiratory muscle strength and therefore represents a clinically meaningful trial endpoint [24]. Further secondary endpoints are Load(cmH20), Power (Watt), Energy (Joule), Volume(l), and sit-to-stand test.

(LOAD) is a measure of resistance to inhalation, and represents the pressure generated in the airways due to the force of the inspiratory muscles during a training session. As the training load decays with increasing lung volume (in order to match the length-tension characteristics of the inspiratory muscles), the load displayed corresponds to the resistance at the start of inhalation (i.e. at RV). A higher load result means that the patient is training their inspiratory muscles harder, leading to stronger muscles. Stronger inspiratory muscles will need to work less hard to cope with the demands of breathing, leading to reduced breathlessness [cmH20].

(POWER) is a measure of muscle performance which combines strength and speed of movement (pressure × flow). More powerful muscles will be more resistant to fatigue at a given level of work and therefore, breathlessness will be reduced. The value displayed is the average power for all breaths in a training session.

(Breathing Energy) is a measure of the mechanical work (or effort) of breathing during your breathing training session. It is a result which combines the force exerted by your inspiratory muscles and the volume (1) of air inhaled. The higher the value of breathing energy you attain, the longer and harder you have worked your inspiratory muscles.

During the sit-to-stand test, the quantity [n] how often a patient can sit down on a chair and stand up during 60 s is noted. These values are evaluated at admission to hospital for surgery and 5 days after surgery.

## Participant timeline {13}

The participant timeline is shown in Table 1.

**Table 1:** Participant timeline

***Visit***	***1***	***2***	***3***	***4***		***6***	***7***	***8***
*Time (day)*		***0***	***7 +/-2d*** ^***a***^	***14 +/-4d***	*0*	***5+/-2d pod***	***x pod***	***90+/-14 pod***
*Patient schedule*	***Screening visit***	***Baseline visit***	***Compliance visit***^a^	***Admission to hospital***	***Surgery***	***Day 5 after surgery***	***End of hospitalisation***	***End of study visit***
*Hospital status*	*Outpatient*				*Inpatient*			*Outpatient clinic*
*Patient information and informed consent*	*x*							
*Demographics*	*x*							
*Medical history*	*x*			*x*				
*Medication*^b^	*x*			*x*			*x*	
*In-/exclusion criteria*	*x*	*x*						
*Administer medical device and training instruction*		*x*						
*Inspiratory muscle training (IMT)*		x	*x*	*x*				
*Power®Breathe device*		*KHP2*^*c*^*,KH2*	*KHP2*	*KHP2*^d^		*KH2*		
*Compliance check and training performance*			*x*					
*VAS/NRS/ Vital signs*				*x*		*x*	*x*	
*BMI*	*x*			*x*				
*Maximum inspiratory pressure*		*x*		*x*		*x*		
*Load (cmH20), Power (Watt), Breathing Energy (Joule), Volume (l)[measured by Power®Breathe device*]		*x*	*x*	*x*		*x*		
*Sit-to-stand test*		*x*		*x*		*x*		
*(Serious) Adverse events*		*x*	*x*	*x*		*x*	*x*	*x*
*Complications*							*x*	*x*

^a^When IMT (inspiratory muscle training) > 15 days, patients are offered an additional appointment with physio on demand, scheduled training can be extended up to 6 weeks

^b^Antibiotics, immunosuppressants, anticoagulants, insulin, oral antidiabetics

^c^Hand out Power®Breathe KHP2 device by a physiotherapist to patient

^d^Return of Power®Breathe KHP2 device to the physiotherapist on the ward

*pod* postoperative day, *VAS* visual analogue scale, *NRS* numerical rating scale, *BMI* body mass index

Control group starts at Visit4: hospital admission. The postoperative procedure is the same for both groups

### Sample size {14}

The sample size calculation was based on:

- A mean Comprehensive Complication Index (CCI) of 30±10 (mean±SD) in the control group, derived from a dataset of patients undergoing upper GI surgery in our department [25].
- A minimal clinically relevant reduction of the CCI by 6 with pre-habilitation, resulting in a CCI of 24±10 (Mean±SD).
- An alpha error of 0.05 (5 %) and a power of 0.9 (90 %) based on a two-sample *t* test to compare the two group means.
- A drop-out rate of 10 %.

The power was calculated for a range of total sample sizes, *n*_*i*=1,.,39_ = 10, ..., 200, using the R [26] package sse [27]. To assess the sensitivity of the sample size calculation with respect to the expected treatment effect, we used a range of effect sizes, θ, from 2 to 10.

**Fig. 1:**
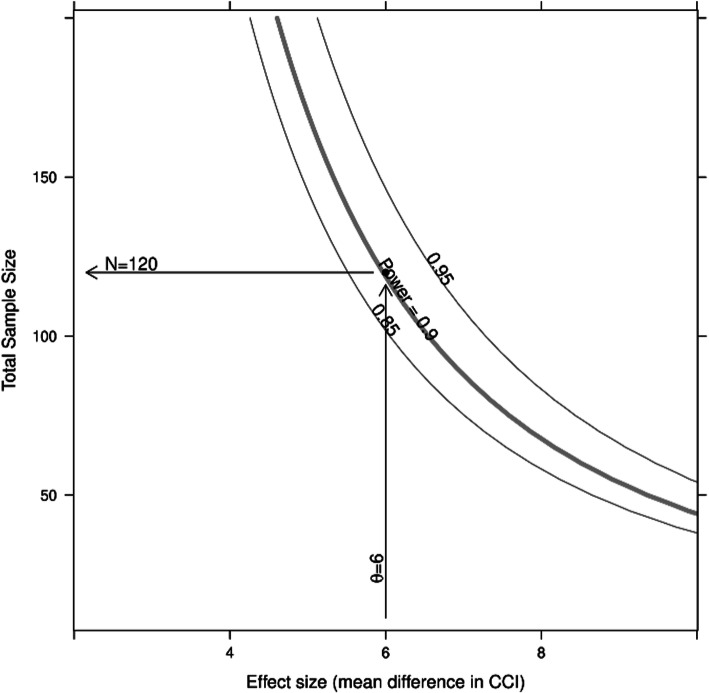
Sensitivity of the sample size with regard to the expected effect size, θ, given a within group standard deviation of 10. An example is shown for θ =6 and a power of 90 %. The curves are smoothed and shown for illustration only

Under the assumptions described above, 134 patients should be recruited for this study (67 per group), to ensure 120 evaluable patients considering a drop-out rate of 10 %. Figure 1 shows how the total sample size (both groups together, without drop-outs) depends on the expected effect size, θ.

### Recruitment {15}

Patients are screened by treating physicians in the outpatient clinic where the indication for elective abdominal surgery is made.

Patients are seen by a physical therapist in order to explain the training in a separate meeting.

## Assignment of interventions: allocation

### Sequence generation {16a}

Consecutively screened and eligible patients will be included in the trial. In order to achieve comparable study groups, patients will be allocated by randomization after given written informed consent using SecuTrial®, conducted at the Clinical Trial Centre (CTC), University of Zürich.

### Concealment mechanism {16b}

Randomization will be performed via variance minimization using the variables patient age and BMI, localization of disease (upper vs. lower gastro-intestinal, HPB, hernia, others) and disease dignity (benign vs. malign). To avoid allocation being fully deterministic, we will set the predictability to 80%.

### Implementation {16c}

The randomization is performed in a 1:1 ratio to either the intervention or the control arm of the study with SecuTrial® at the CTC at the University of Zürich.

Enrolling of patients will be performed by DB, KP or AM.

## Assignment of interventions: blinding

### Who will be blinded {17a}

The participants as well as the treating persons will not be blinded. The further treatment is not influenced by the preoperative respiratory training and study objectives are all objective measurements and not dependent on the treating physicians, as they are registered in the Power®Breathe KHP2 device. The outcome assessors are partially blinded and the analysis will be performed by a blinded statistician.

### Procedure for unblinding if needed {17b}

The design is open label with outcome assessors being partially blinded and a blinded data analyst so unblinding will not occur.

## Data collection and management

### Plans for assessment and collection of outcomes {18a}

The following documents are considered source data, including but not limited to:

- The main source of information is our clinic information system.
- SAE worksheets.
- Nurse records, records of clinical coordinators and physiotherapists.
- Medical records from other department(s), or other hospital(s), or discharge letters and correspondence with other departments/hospitals, if the participant visited any during the study period and the post-study period.

The investigators will use electronic case report forms (eCRF) of SecuTrial®, one for each enrolled study participant, to be filled in with all relevant data pertaining to the participant during the study. All participants who either entered the study or were considered not eligible or were eligible but not enrolled into the study additionally must be documented on a screening log. The investigator will document the participation of each study participant on the enrolment Log.

### Plans to promote participant retention and complete follow-up {18b}

Patients are encouraged to adhere to the training program as well as to perform physical activity besides the inspiratory training. Correspondingly, healthcare professionals are able to review patient progress by tracking up to 36 training sessions, which the KHP2 can store. Clinical research has shown high patient motivation due to the on-screen feedback which has resulted in high compliance (90%+) and significantly improved lung muscle strength and stamina.

### Data management {19}

For data and query management, monitoring, reporting and coding an internet-based secure data base SecuTrial® developed in agreement to the Good Clinical Practice (GCP) guidelines provided by the (CTC) Zurich will be used for this study. It is the responsibility of the investigator to assure that all data during the study will be entered completely and correctly in the respective database. Corrections in the eCRF may only be done by the investigator or by other authorized persons. In case of corrections, the original data entries will be archived in the system and can be made visible. For all data entries and corrections date, time of day and person who is performing the entries will be generated automatically.

The trial master file present at the University Hospital of Zürich will have all requested original data filed.

### Confidentiality {27}

It is assured that any authorized person, who may perform data entries and changes in the eCRF, can be identified. A list with signatures and initials of all authorized persons will be filed in the study site file and the trial master file, respectively.

Documented medical histories and narrative statements relative to the participants’ progress during the study will be maintained in the clinical information system. These records will also include the following: originals or copies of laboratory and other medical test results (e.g. ECGs, etc.) which must be kept on file with the individual participant’s *eCRF*.

The investigators assure to perform a complete and accurate documentation of the participant data in the eCRF. All data entered in the eCRF must also be available in the individual participant file either as printouts or as notes taken by either the investigator or another responsible person assigned by the investigator.

Essential documents must be retained for at least 10 years after the regular end or a premature termination of the respective study (KlinV Art. 45). Essential documents must be retained according to local law in the case of international multicentre studies.

Direct access to source documents will be permitted for purposes of monitoring, audits and inspections.

### Plans for collection, laboratory evaluation and storage of biological specimens for genetic or molecular analysis in this trial/future use {33}

All study data will be archived for 10 years after study termination or premature termination of the clinical trial. On the consent form, participants will be asked if they agree to use of their data should they choose to withdraw from the trial. Participants will also be asked for permission for the research team to share relevant data with people from the Universities taking part in the research or from regulatory authorities, where relevant. This trial does not involve collecting biological specimens for storage.

## Statistical methods

### Statistical methods for primary and secondary outcomes {20a}

The full analysis set (FAS) will include all patients randomized for this trial. The FAS will be analysed according to the intention-to-treat (ITT) principle, analysing patients according to the randomly allocated treatment. The per-protocol set (PPS) will include all patients from the FAS without major protocol deviations and with minimum compliance regarding the trainings. The PPS will be analysed “as treated”, analysing patients according to the received treatment.

The primary outcome (CCI) at 90 days after surgery will be compared between treatment groups using a linear model with treatment as explanatory factor and age, BMI, disease localization (upper vs. lower gastro-intestinal, HPB, hernia, others) and disease dignity as covariates (variables used for minimization in the randomization process). In case of violation of the normality assumption, log-transformation of the primary outcome will be considered. The estimate of the treatment effect will be reported with a 95 % confidence interval. The main analysis will be a complete case analysis applied to the FAS.

To assess the robustness of the result, the following sensitivity analyses will be performed:

- A two-sample *t* test to compare the treatment groups
- A non-parametric Wilcoxon rank-sum test to compare the treatment groups
- A covariate-adjusted analysis as the main analysis described above, but with more risk factors for postoperative complications as covariates

The main analysis and the sensitivity analyses listed above are first applied to the FAS (analysed by intention-to-treat).

CCI at the end of hospitalization will be analysed as the primary outcome (main analysis). Postoperative morbidity, assessed by the Clavien-Dindo classification (maximum grade per patient), will be compared between treatment arms with a Wilcoxon rank-sum test and using a logistic regression model on a dichotomized version of the Clavien-Dindo classification (e.g., major complications, grade ≥ 3b).

The length of stay (LOS) will be analysed as time to hospital discharge alive, using a Cox proportional hazards model. If there are patients who died during the hospital stay, in-hospital death will be considered as competing risk and patients will be censored at the date of death in a cause-specific Cox proportional hazards model. The binary outcomes readmission and mortality within 90 days will be compared with a logistic regression model. MIP, Load, Energy, Power, Volume and the sit-to-stand test results, which are measured multiple times per patient, will be compared with linear mixed-effects models with a random intercept per patient to account for the non-independence of repeated measurements on the same patient. Treatment will be used as explanatory factor with age, BMI, disease localization and disease dignity as covariates in the statistical models as in the analysis of the primary outcome. In addition, time of measurement and the interaction of time and treatment will be included in the mixed-effects models. Secondary outcomes will be evaluated using complete case analysis applied to the FAS.

### Interim analyses {21b}

There are no interim analyses planned.

### Methods for additional analyses (e.g. subgroup analyses) {20b}

The following subgroups will be investigated for their potential to alter the effect of pre-habilitation on the primary outcome:

- Localization of the disease (upper vs. lower gastro-intestinal, HPB, hernia, others)
- Disease dignity (benign vs. malign)
- BMI
- Age

Each subgroup variable will be added to the statistical model described for the main analysis above, together with the interaction of the subgroup variable with treatment. A significant interaction will indicate that the treatment effect depends on the subgroup.

### Methods in analysis to handle protocol non-adherence and any statistical methods to handle missing data {20c}

All or a subset of the above analyses are applied to the PPS (analysed as treated) to address the problem of non-adherence to treatment. If missing outcome measurements are present, we will do another series of sensitivity analyses using multiple imputation to assess whether missing outcome measurements may have biassed the results. All imputed datasets will be analysed by the methods described above and results will be pooled using Rubin’s rules [28].

### Plans to give access to the full protocol, participant-level data and statistical code {31c}

The full protocol is available from the corresponding author on request and will be published alongside the results report as supplementary material.

## Oversight and monitoring

### Composition of the coordinating centre and trial steering committee {5d}

For data and query management, monitoring, reporting and coding an internet-based secure data base SecuTrial® developed in agreement to the Good Clinical Practice (GCP) guidelines provided by the (CTC) Zurich will be used for this study. It is the responsibility of the investigator to assure that all data during the study will be entered completely and correctly in the respective database. Corrections in the eCRF may only be done by the investigator or by other authorized persons. In case of corrections, the original data entries will be archived in the system and can be made visible. For all data entries and corrections date, time of day and person who is performing the entries will be generated automatically

### Composition of the data monitoring committee, its role and reporting structure {21a}

Monitoring will be performed internally. Service of the CTC include project start planning, basic monitoring visits, quality controls and supervision.

All original data including all patient files, progress notes and copies of laboratory and medical test results will be available for monitoring.

### Adverse event reporting and harms {22}

The investigator has the responsibility for SAE identification, documentation, and assessing the causal relationship study intervention.

All SAEs will be fully documented in the appropriate *eCRF*. For each SAE, the investigator will provide the onset, duration, treatment required, outcome and action taken with regard to the study intervention.

If, during a clinical trial, serious adverse events occur in participants in Switzerland, and it cannot be excluded that the events are attributable to the intervention under investigation, the investigator must report these events:

- To the CEC within 15 days.

If immediate safety and protective measures (with possible relation to the POWER®Breathe device) have to be taken during the conduct of this clinical trial, the investigator must notify the CEC of these measures, and of the circumstances necessitating them, within 7 days.

Participants terminating the study (either regularly or prematurely) with the following will return for a follow-up investigation:

- Reported ongoing SAE, or
- Any ongoing AEs of laboratory values or of vital signs being beyond the alert limit

This visit will take place up to 30 days after terminating the treatment period. Follow-up information on the outcome will be recorded on the respective SAE page in the eCRF.

Follow-up investigations may also be necessary according to the investigator’s medical judgement even if the participant has no SAE at the end of the study. However, information related to these investigations does not have to be documented in the eCRF but must be noted in the source documents.

### Frequency and plans for auditing trial conduct {23}

A quality assurance audit/inspection of this study may be conducted by the CEC. The quality assurance auditor/inspector will have access to all medical records, the investigator’s study-related files and correspondence, and the informed consent documentation that is relevant to this clinical study.

The investigator will allow the persons being responsible for the audit or the inspection to have access to the source data/documents and to answer any questions arising. All involved parties will keep the patient data strictly confidential.

### Plans for communicating important protocol amendments to relevant parties (e.g. trial participants, ethical committees) {25}

Substantial amendments (significant changes) are only implemented after approval of the CEC.

Significant changes to be authorized by the CEC are the following:

- Changes affecting the participants’ safety and health, or their rights and obligations;
- Changes to the protocol, and in particular changes based on new scientific knowledge which concern the trial design, the method of investigation, the endpoints or the form of statistical analysis;
- A change of trial site, or conducting the clinical trial at an additional site; or
- A change of sponsor, coordinating investigator or investigator responsible at a trial site.

Under emergency circumstances, deviations from the protocol to protect the rights, safety and well-being of human participants may proceed without prior approval of the sponsor and the CEC. Such deviations shall be documented and reported to the CEC as soon as possible.

All non-substantial amendments are communicated to the CEC within the Annual Safety Report (ASR).

### Dissemination plans {31a}

After the statistical analysis of this trial, the sponsor will make every endeavour to publish the data in a medical journal.

## Discussion

One of the main problems with the term “prehabilitation” is its definition. “Prehabilitation” means exercise, nutrition, and psychosocial interventions equally and often in combination to optimize the baseline health status before surgery. In fact, any intervention before surgery can be meant by the term; it encompasses more than a simple switch of rehabilitation to the preoperative schedule.

Any general statement that pre-rehabilitation helps to prevent postoperative complications is therefore abundantly imprecise. It remains unclear whether one of the various interventions alone is sufficient and should then be used on a larger scale. It is equally unclear whether, and if so, which intervention can be dispensed with.

Twenty years ago, one of the first RCTs on prehabilitation showed a shorter hospital stay and less time on the intensive care unit in patients undergoing coronary artery bypass surgery after a preoperative program consisting of training, education and re-inforcement [29]. Although this is important data, it does highlight some of the difficulties we still face in prehabilitation studies today.

Patients trained for a minimum of 10 weeks before surgery [29]. However, from this, a general conclusion cannot be drawn that prehabilitation shortens the inpatient stay. Oncological operations can hardly be planned long in advance, so any prehabilitation intervention must be able to achieve the greatest possible effect in the shortest possible time, esp. in these patients.

Secondly, combined interventions are to be viewed critically insofar as they are realised under study conditions with a high organisational and personnel effort. These studies consist of multi-stage concepts that are comprehensively supervised, but whose implementation in clinical practice is hardly possible. There is a clear need for randomized-controlled studies with a simple protocol that can be easily transferred into clinical practice.

A third problem is the translation of physiotherapy data into clinically relevant outcomes, i.e. postoperative complications or hospital stay. Physiotherapy measurements are not causally related to postoperative outcomes, they are at most correlated, although the degree of correlation is not entirely clear. A recent randomized trial found that an exercise prehabilitation program on a cycle ergometer supervised by a physical therapist created a significant increase of oxygen uptake (VO_2_) at the ventilatory anaerobic threshold [30].

There is one cohort study that shows a significant correlation between VO_2_ and postoperative morbidity, but in the same study, the correlation also exists against BMI and age [31]. A multivariate analysis that would have explicitly examined the value of VO_2_ was not conducted in this study and therefore an improvement in VO_2_ cannot be directly inferred to a lower complication rate. So far, the focus on pneumological measures seems inconclusive in terms of their impact on postoperative outcomes. It is exactly for this reason, we collect physiotherapy and pneumology data in our study, but the primary endpoint is postoperative complications. Postoperative complications are the sole reason we do prehabilitation, so in principle, it should be the end point.

A second important fact of the study is the effortless adaptation of standard respiratory physiotherapy from currently postoperative to preoperative. The respiratory training carried out in our study corresponds to postoperative rehabilitation training, where it is a proven tool. Therefore, physiotherapy can apply such a concept without needing special training. Thirdly, the external measurement by the device eliminates the diary listing of patients’ performances and allows the exercise adherence and thus the effect to be objectively recorded. Thus, the study explores a patient-centred training approach, whereby the digital recording of training sessions and results allows the individual adherence of the subjects to the training to be accurately recorded. This eliminates self-assessment with all its disadvantages.

We hope that the study presented here will provide valid information on the effects of pragmatic preoperative inspiratory therapy on postoperative complications after major abdominal surgery.

## Trial status

Version 2.0; July 17th, 2021; approved by the local ethics committee.

This trial is recruiting, the first patient was enrolled on August 13th, 2021.

Registered August 13^th^, 2020, ClinicalTrials.gov: NCT 04558151, https://clinicaltrials.gov/ct2/show/NCT04558151?term=Birrer&cntry=CH&draw=2&rank=1

Approximate date of recruitment completion: 12/2023

## Abbreviations

ASR: Annual Safety Report
BASEC: Business Administration System for Ethical Committees
CA: Competent Authority (Swissmedic)
CEC: Cantonal Ethics Committee (canton: member state of Swiss Confederation)
CCI: Comprehensive Complication Index
ClinO: Clinical Trial Ordinance (KlinV)
COPD: Chronic obstructive pulmonary disease
CTC: Clinical trial centre
eCRF: Electronic case report form
FAS: Full analysis set
FEV1: Forced expiratory volume in 1 s
GCP: Good Clinical Practice
ICH: International Council for Harmonisation of Technical Requirements for Pharmaceuticals for Human Use
ITT: Intention to treat
KlinV: Verordnung über klinische Versuche (Clinical Trial Ordinance)
LOS: Length of stay
MIP: maximal inspiration pressure (MIP)
PI: Principal Investigator
PPS: Per protocol set
SAE: Serious Adverse Event
SDV: Source data verification
SNCTP: Swiss National Clinical Trial Portal
SOP: Standard operating procedure
TMF: Trial master file
VAS/NRS: Visual analogue scale/numeric rating scale
VC: Vital capacity [litre; l]
